# *microshades*: An R Package for Improving Color Accessibility and Organization of Microbiome Data

**DOI:** 10.1128/mra.00795-22

**Published:** 2022-10-31

**Authors:** Erin M. Dahl, Emory Neer, Kate R. Bowie, Eric T. Leung, Lisa Karstens

**Affiliations:** a Department of Medical Informatics and Clinical Epidemiology, Division of Bioinformatics and Computational Biomedicine, Oregon Health & Science University, Portland, Oregon, USA; b Knight Cancer Institute, Cancer Early Detection Advanced Research Center, Oregon Health & Science University, Portland, Oregon, USA; Indiana University, Bloomington

## Abstract

When creating figures, it is important to consider that individuals with color vision deficiency (CVD) may not perceive all colors. While there are several CVD-friendly color palettes, they are often insufficient for working with microbiome data. Here, we introduce *microshades*, an R package for creating CVD-accessible microbiome figures.

## ANNOUNCEMENT

Color vision deficiency (CVD), commonly known as colorblindness, affects 1 in 12 men and 1 in 200 women, approximately 300 million people worldwide (1). Individuals with CVD do not experience complete loss of color vision but have reduced ability to distinguish between different colors. There are three common types of CVD, i.e., deuteranopia, protanopia, and tritanopia. Individuals with deuteranopia (red-green colorblindness) have difficulty distinguishing between shades of red, green, and yellow. Individuals with protanopia (red colorblindness) have diminished ability to distinguish between colors containing red, whereas those with tritanopia have difficulty distinguishing between blues and yellows.

Despite the large numbers of individuals who experience CVD, many scientific figures rely on color to convey information. To ensure that colors in figures are accessible to all, researchers can use colorblind-friendly color palettes and evaluate accessibility with colorblind simulators. There are several CVD-friendly color palettes available (2–5); however, they are typically limited to 8 to 15 distinct colors or may not be accessible for all forms of CVD. Even with these resources, it can be challenging to apply these schemes to scientific figures. For example, for visualization of microbiome data it is common to represent tens to hundreds of bacterial taxa in one figure, for which the currently available CVD color palettes are insufficient.

**FIG 1:**
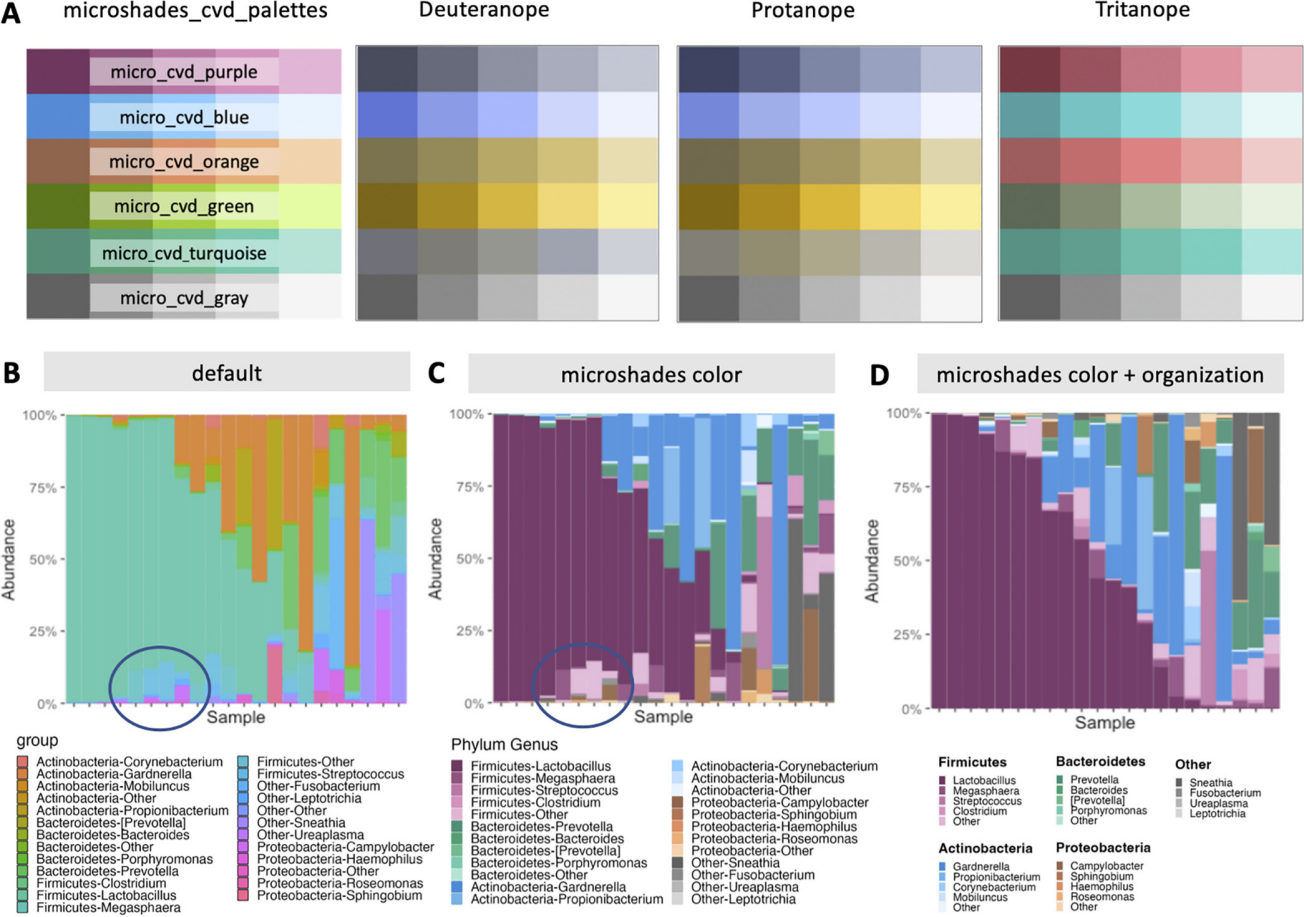
(A) The *microshades* universal CVD-friendly palette, microshades_cvd_palettes, with CVD emulations for deuteranope, protanope, and tritanope accessibility. The cvdemulator (http://hclwizard.org:3000/cvdemulator) was used. (B to D) Example of application to a stacked barplot representing microbiome data (7). In each plot, columns represent samples and the colors represent different bacteria within each sample. The blue circles in B and C highlight the visual improvement that results from using the *microshades* colors. (B) In the default plot, colors are determined by the default ggplot2 color scheme. (C) The *microshades* color plot contains the same data and in the same sample order, with the microshades_cvd_palettes applied. Colors are organized to represent the phylum-genus classification of bacteria found in the samples. (D) The *microshades* color plus organization plot uses the microshades_cvd_palettes, *microshades* organizational functions, and *microshades* custom legend.

To overcome this limitation, we developed the *microshades* R package, which provides custom color shading palettes to improve CVD accessibility and data organization. The *microshades* package includes two color palettes, namely, microshades_cvd_palettes and microshades_palettes (Table 1). To construct these palettes, hue (type of color), chroma (colorfulness), and luminance (brightness) were adjusted for optimal visual distinction and CVD accessibility. Each color palette contains six hues with five sequential variations of chroma and luminance per hue, for a total of 30 available colors per palette. All shades have been tested with a CVD simulator, cvdemulator (3), for deuteranope, protanope, and tritanope accessibility (Fig. 1A).

**TABLE 1:**
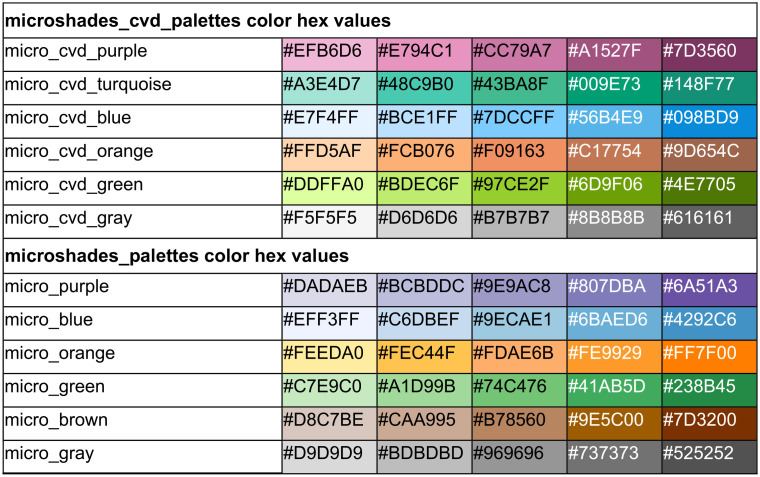
Colors in the palettes available in *microshades*

In addition to color palettes, the *microshades* package contains functions to aid in the complex data visualization common in microbiome studies (Fig. 1B to D), including functions to group data by taxonomic ranking. For example, hues correspond to a high-order taxonomic group (e.g., phylum) and shades of each hue can represent subgroups of the taxonomic group (e.g., genus). Subgroup shading is determined by abundance in the data set, with darker shades indicating the most abundant subgroups, and less abundant subgroups are collapsed into an “other” category. There are also functions to aid in data organization, such as vertical and horizontal sorting of the data and restructuring of the plot legends. Contribution plots can also be created, providing greater insight by displaying boxplots, median barplots, or mean barplots for individual taxa.

In summary, the *microshades* R package is a visualization tool for microbiome researchers. The package contains two CVD-accessible palettes, along with several organization features. The *microshades* package can be used in conjunction with common microbiome R packages, such as phyloseq (6), to enhance microbiome data visualization.

### Data availability.

*microshades*, along with microbiome and nonmicrobiome examples using *microshades*, is available for download at https://karstenslab.github.io/microshades.
